# A new species of *Linan* Hlavácˇ (Coleoptera, Staphylinidae, Pselaphinae) from Shenzhen, China

**DOI:** 10.3897/zookeys.859.35465

**Published:** 2019-07-02

**Authors:** Qing-Hao Zhao, Wang Xu, Zi-Wei Yin

**Affiliations:** 1 College of Life Sciences, Shanghai Normal University, Shanghai 200234, China Shanghai Normal University Shanghai China; 2 Shenzhen Environmental Monitoring Center, Shenzhen 518049, Guangdong, China Shenzhen Environmental Monitoring Center Shenzhen China

**Keywords:** Ant-loving beetles, southern China, taxonomy, Tyrini

## Abstract

A new Chinese species of the genus *Linan* Hlaváč, 2003, *L.qiniangmontis***sp. nov.**, is described based on two male and three female specimens from sifted leaf litter samples at Qiniang Mountain, Shenzhen City, Guangdong. The species can be readily recognized and separated from all congeners based on the forms of the male antennae, the metaventral processes, and the aedeagus.

## Introduction

The Oriental genus *Linan* Hlaváč, 2003 belonging to the ‘*Pselaphodes* complex’ of genera (Hlaváč 2003; Yin et al. 2013a) is a small group containing 16 species distributed in China (16 spp.) and Thailand (1 sp.) (Hlaváč 2003; Yin et al. 2011, 2013b; Yin and Li 2012, 2013; Zhang et al. 2018). An identification key and distributional maps of the genus were recently provided by Zhang et al. (2018). A survey of the local coleopterous fauna in Shenzhen City has resulted in the discovery of the 17^th^ species of *Linan*, which is described here.

## Materials and methods

The material used in this paper is housed in the Insect Collection of Shanghai Normal University, Shanghai, China (**SNUC**). The text of the specimen labels is quoted verbatim, with original Chinese names listed in parentheses.

Dissected parts were preserved in Euparal on plastic slides that were placed on the same pins as the respective specimens. The habitus images were taken using a Canon 5D Mark III camera with a Canon MP-E 65mm f/2.8 1–5X Macro Lens, and a Canon MT-24EX Macro Twin Lite Flash used as the light source. Images of the morphological details were produced using a Canon G9 camera mounted to an Olympus CX31 microscope under transmitted light. Zerene Stacker (version 1.04) was used for image stacking. All images were modified and grouped into plates in Adobe Photoshop CS5 Extended.

The abdominal tergites and sternites are numbered following Chandler (2001) in Arabic (starting from the first visible segment) and Roman (reflecting true morphological position) numerals, e.g., tergite 1 (IV), or sternite 7 (IX).

## Taxonomy

### Linan qiniangmontis sp. nov.

Taxon classificationAnimaliaColeopteraStaphylinidae

http://zoobank.org/122313A9-AF98-429B-BBBE-D13402160D5C

Figs 1 , 2

#### Type material.

**Holotype: CHINA**: ♂: ‘China: Guangdong, Shenzhen City, Mt. Qiniang (七娘山), 23°32'28.73"N, 114°35'8.46"E, mixed leaf litter, sifted, 45 m, 23.III.2019, Tang, Shuai, Zhao, Zhou & Xia leg.’ (SNUC). **Paratypes: CHINA**: 1 ♂, 3 ♀♀, same label data as holotype (SNUC).

#### Diagnosis.

Body length slightly less than 2.5 mm. Male: antennal club almost simple, with antennomere IX slightly angulate at anteromesal corner; metaventral processes short and narrowing toward apex; protibiae with small denticle at apex; metacoxae with truncate, curved, ventral projection; aedeagus elongate, median lobe asymmetrically narrowed at apex. Female: identifiable only when in association with a male.

#### Description.

**Figure 1.**
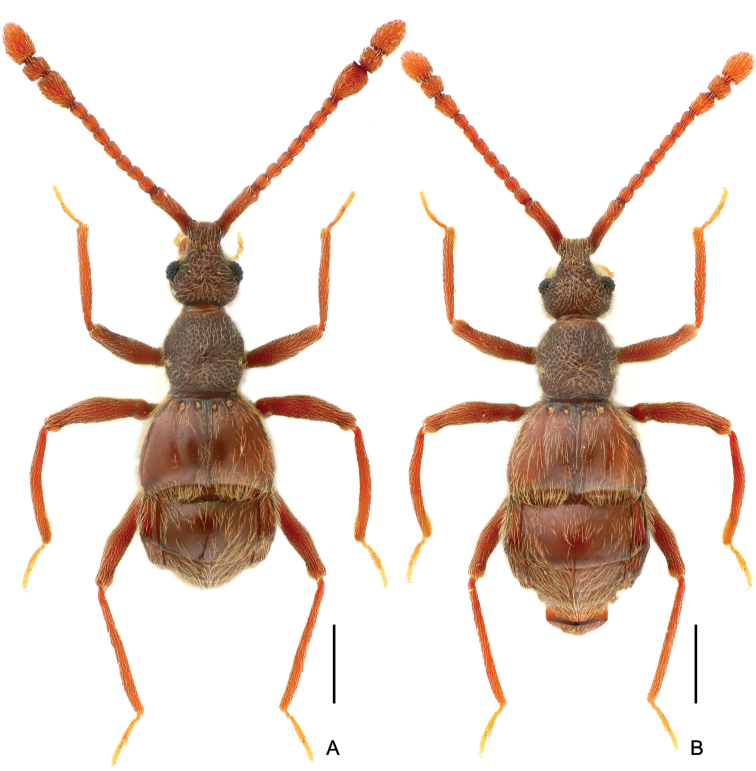
Dorsal habitus of *Linanqiniangmontis* sp. nov. **A** male **B** female. Scale bars: 0.5 mm.

**Figure 2.**
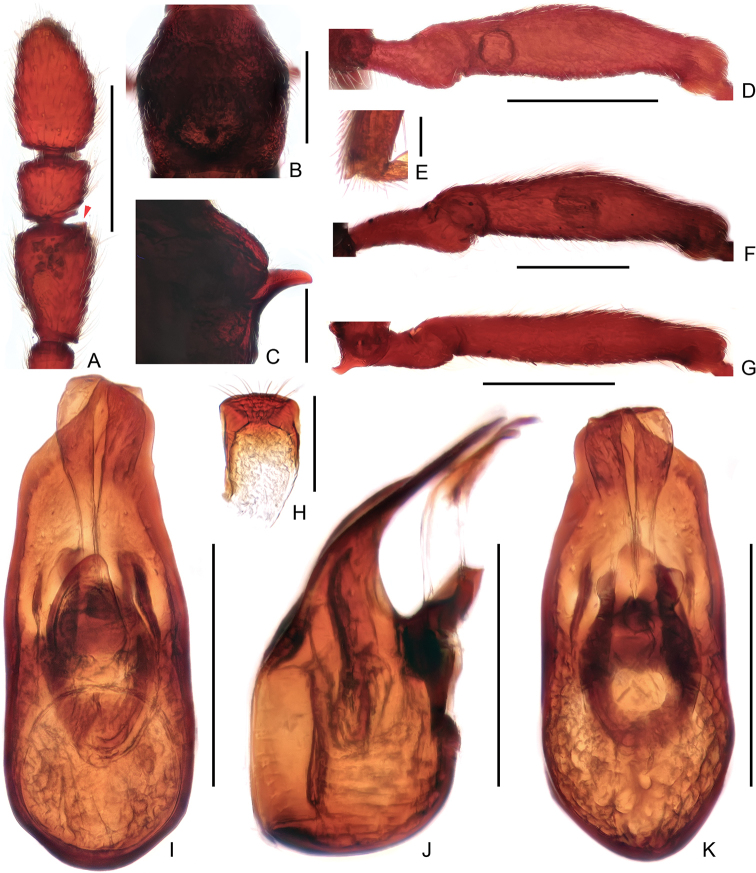
Male diagnostic features of *Linanqiniangmontis* sp. nov. **A** antennal club **B** pronotum **C** metaventral process, lateral **D** protrochanter and profemur **E** apex of protibia **F** mesotrochanter and mesofemur **G** metacoxa, metatrochanter, and metafemur **H** sternite IX **I–K** aedeagus, dorsal (**I**), lateral (**J**), and ventral (**K**). Scale bars: 0.3 mm (**A, B, D, F, G**); 0.2 mm (**C, I, J, K**); 0.1 mm (**H**); 0.05 mm (**E**).

Male (Fig. 1A). Body length (combined length of head, pronotum, elytra, and abdomen) 2.32–2.33 mm. Head longer than wide, length from clypeal anterior margin to head base 0.52–0.54 mm, width across eyes 0.48–0.49 mm; eyes small, each composed of ca. 23 facets. Antennae elongate, 1.78–1.79 mm long, scape elongate, ca. 3.5 times as long as wide, antennomeres 2–8 each sub-moniliform, of similar width, antennal club (Fig. 2A) formed by antennomeres 9–11, antennomere 9 much longer than wide, broadening from base to apex, angulate at anteromesal corner (Fig. 2A, indicated by arrow), antennomere 10 slightly transverse, antennomere 11 truncate and broadest at base and narrowing apically, both antennomeres 10 and 11 simple. Pronotum (Fig. 2B) approximately as long as wide, with rounded lateral margins, length along midline 0.49–0.51 mm, maximum width 0.49–0.52 mm. Elytra strongly transverse, length along suture 0.56–0.57 mm, maximum width 0.85–0.88 mm. Metaventral processes (Fig. 2C) short, narrowing apically. Protrochanters and profemora (Fig. 2D) simple, protibiae (Fig. 2E) with small but distinct denticle at apex; mesotrochanters, mesofemora, and mesotibiae (Fig. 2F) simple; metacoxae (Fig. 2G) with truncate curved projection on ventral margin; metatrochanters, metafemora, and metatibiae simple. Abdomen approximately as wide as elytra, length of dorsally visible part along midline 0.74–0.77 mm, maximum width 0.86–0.87 mm; tergite 1 (IV) more than twice as long as tergite 2 (V); sternite 7 (IX) (Fig. 2H) semi-membranous, elongate. Length of aedeagus (Fig. 2I–K) 0.38–0.40 mm; median lobe asymmetrical dorso-ventrally, narrowing apically with pointed apex; elongate parameres slightly exceeding apex of median lobe, with rounded apices; endophallus with one broad, rounded triangular sclerite, and one much shorter, elongate sclerite forked at apex.

Female. Similar to male in general morphology, with slightly shorter antennae and smaller eyes; antennae and legs simple; lacking metaventral processes. Eyes each composed of approximately 18 facets. Measurements (as of male): Body length 2.33–2.44 mm, length/width of head 0.53–0.55 /0.49–0.51 mm, length of antennae 1.63–1.70 mm, length/width of pronotum 0.50–0.51/0.51 mm, length/width of elytra 0.57/0.89–0.91 mm, length/width of abdomen 0.72–0.81/0.92–0.93 mm.

#### Distribution.

China: Guangdong.

#### Etymology.

The new species epithet refers to the type locality of the new species, Qiniang Mountain.

#### Comparative notes.

The new species is placed as a member of the *L.chinensis* group by the almost unmodified antennal clubs in the male. It is most similar to *L.hujiayaoi* Yin & Li, 2013 and *L.mulunensis* Zhang, Li & Yin, 2018 (both from Guangxi) in sharing modified male metacoxae. *Linanqiniangmontis* differs from both known species in the slightly angulate anteromesal corner of antennomere 9 (rounded in *L.hujiayaoi* and *L.mulunensis*), a different form of the metaventral processes (processes stouter in *L.hujiayaoi* and much more elongate in *L.mulunensis*), the lack of additional projections above the metacoxae (present in *L.hujiayaoi* and *L.mulunensis*), and a more elongate aedeagus with a different configuration of the endophallus.

## Supplementary Material

XML Treatment for Linan qiniangmontis
